# Dominant Mutations in *S. cerevisiae PMS1* Identify the Mlh1-Pms1 Endonuclease Active Site and an Exonuclease 1-Independent Mismatch Repair Pathway

**DOI:** 10.1371/journal.pgen.1003869

**Published:** 2013-10-31

**Authors:** Catherine E. Smith, Marc L. Mendillo, Nikki Bowen, Hans Hombauer, Christopher S. Campbell, Arshad Desai, Christopher D. Putnam, Richard D. Kolodner

**Affiliations:** 1 Ludwig Institute for Cancer Research, University of California School of Medicine, San Diego, La Jolla, California, United States of America; 2 Department of Cellular and Molecular Medicine and Moores-UCSD Cancer Center, University of California School of Medicine, San Diego, La Jolla, California, United States of America; 3 Department of Medicine, University of California School of Medicine, San Diego, La Jolla, California, United States of America; 4 Institute of Genomic Medicine, University of California School of Medicine, San Diego, La Jolla, California, United States of America; Duke University, United States of America

## Abstract

Lynch syndrome (hereditary nonpolypsis colorectal cancer or HNPCC) is a common cancer predisposition syndrome. Predisposition to cancer in this syndrome results from increased accumulation of mutations due to defective mismatch repair (MMR) caused by a mutation in one of the mismatch repair genes *MLH1*, *MSH2*, *MSH6* or *PMS2/scPMS1*. To better understand the function of Mlh1-Pms1 in MMR, we used *Saccharomyces cerevisiae* to identify six *pms1* mutations (*pms1-G683E*, *pms1-C817R*, *pms1-C848S*, *pms1-H850R*, *pms1-H703A* and *pms1-E707A*) that were weakly dominant in wild-type cells, which surprisingly caused a strong MMR defect when present on low copy plasmids in an *exo1Δ* mutant. Molecular modeling showed these mutations caused amino acid substitutions in the metal coordination pocket of the Pms1 endonuclease active site and biochemical studies showed that they inactivated the endonuclease activity. This model of Mlh1-Pms1 suggested that the Mlh1-FERC motif contributes to the endonuclease active site. Consistent with this, the *mlh1-E767stp* mutation caused both MMR and endonuclease defects similar to those caused by the dominant *pms1* mutations whereas mutations affecting the predicted metal coordinating residue Mlh1-C769 had no effect. These studies establish that the Mlh1-Pms1 endonuclease is required for MMR in a previously uncharacterized Exo1-independent MMR pathway.

## Author Summary

Lynch syndrome (hereditary nonpolypsis colorectal cancer or HNPCC) is a common cancer predisposition syndrome. Predisposition to cancer in this syndrome results from increased accumulation of mutations due to defective mismatch repair (MMR) caused by a mutation in one of the mismatch repair genes *MLH1*, *MSH2*, *MSH6* or *PMS2/scPMS1*. In addition to these genes, various DNA replication factors and the excision factor *EXO1* function in the repair of damaged DNA by the MMR pathway. Although *EXO1* is considered to be the major repair nuclease functioning in mismatch repair, the relatively low mutation rates caused by an *exo1* deletion suggest otherwise. Here we used genetics, microscopy and protein biochemistry to analyze the model organism *Saccharomyces cerevisiae* to further characterize a poorly understood mismatch repair pathway that functions in the absence of *EXO1* that is highly dependent on the Mlh1-Pms1 complex. Surprisingly, we found that the highly conserved metal binding site that is critical for the endonuclease activity of the Mlh1-Pms1 heterodimer is required for MMR in the absence of Exo1 to a much greater extent than in the presence of Exo1. Thus, this work establishes that there are at least two different polynucleotide excision pathways that function in MMR.

## Introduction

DNA mismatch repair (MMR) acts to repair the potentially mutagenic misincorporation errors that occur during normal DNA replication and the absence of MMR results in increased rates of accumulating mutations. Consequently, defects in human MMR genes cause the hereditary cancer susceptibility syndrome HNPCC (hereditary nonpolypsis colorectal cancer, otherwise known as Lynch syndrome) [1], [2], and loss of MMR function also appears to underlie the development of some sporadic cancers [3]–[7]. MMR also repairs mispaired bases that occur in recombination intermediates as well as prevents inappropriate recombination between DNAs with imperfect homology where recombination could result in genome rearrangements [8]–[10].

The mechanism of MMR has been extensively characterized in both *E. coli* and different eukaryotic systems, with *E. coli* MMR being the best characterized [11]–[14]. In *E. coli* MMR, mismatches are recognized by the MutS homodimer [15], [16]. Mispair bound MutS then recruits the MutL homodimer [17]. This recruitment leads to activation of the MutH endonuclease, which introduces single strand breaks, called nicks, at unmethylated GATC sites in the newly replicated and hemimethylated DNA strand [18]. Next, a combination of the UvrD helicase and one of four single stranded DNA specific exonucleases excise the nicked strand past the mispair and the resulting singled-stranded gap is filled in by DNA polymerase III, single strand DNA binding protein and DNA ligase [14], [19].

In eukaryotes mispairs are recognized by either Msh2-Msh6 or Msh2-Msh3, two partially redundant heterodimers of MutS family member proteins [12], [20], [21]. Mispair bound Msh2-Msh6 and Msh2-Msh3 recruit the MutL related complex, called Mlh1-Pms1 in *S. cerevisiae* and Mlh1-Pms2 in human and mouse [11], [12], [22]–[24]. The Pms1/Pms2 subunit of the Mlh1-Pms1/Pms2 complex is known to contain an endonuclease active site, suggesting that Mlh1-Pms1/Pms2 may be analogous to a combination of both *E. coli* MutL and MutH [25], [26]. Exo1, a DNA exonuclease from the Rad2/XPG family, has been implicated in the excision step of eukaryotic MMR; however, mutations in *S. cerevisiae* and mouse *EXO1* only result in partial MMR defects, suggesting the existence of additional excision mechanisms [27]–[29]. Genetic and biochemical studies have also implicated DNA polymerase δ, RPA, RFC and PCNA in MMR [12], [30]–[37] and have suggested that several of these proteins including PCNA and RFC may function both prior to excision and in the resynthesis steps of MMR [25], [26], [33], [38].

MMR is spatially and temporally coupled to replication *in vivo* [38], [39], providing a mechanism to bring MMR proteins into the proximity of newly formed mispairs. DNA replication generates nicks in the nascent DNA strands that may be involved in MMR [30], [34], [40], [41], consistent with the observation that discontinuous lagging strand MMR is more dependent on excision catalyzed by Exo1 than leading strand MMR [38], [42]. Furthermore, preexisting nicks in DNA target the Mlh1-Pms1/Pms2 endonuclease to the nicked strand *in vitro* [43]. However, these results raise two unresolved questions: Why is it necessary to target additional nicks to an already nicked DNA strand, and if preexisting nicks can support MMR *in vitro* then why is Mlh1-Pms1 absolutely required for MMR *in vivo*? Part of the answer to the apparent contradictions implied by these experimental results could be the presence of multiple MMR pathways in which the same MMR proteins have differing roles. Consistent with this, biochemical studies have identified two types of excision mechanisms that may function in MMR, excision by Exo1 [44] and strand displacement synthesis toward the mispair by DNA polymerase δ potentially coupled with flap cleavage [45]. Both mechanisms could act at either a pre-existing 5′ nick or a 5′ nick introduced by the Mlh1-Pms1/Pms2 endonuclease. Genetic studies have also identified Exo1-dependent and -independent MMR pathways [27]. The Exo1-independent pathway requires the PCNA-Msh2-Msh6 interaction and the Pol32 subunit of DNA polymerase δ and is inactivated by separation-of-function mutations affecting Mlh1, Pms1, Msh2, Msh3, and PCNA that do not affect Exo1-dependent MMR [27], [38]. How these mutations specifically affect in the Exo1-independent MMR pathway and how this pathway excises the nascent DNA strand is unclear.

To better understand the role of both Mlh1-Pms1 and Exo1 in MMR, we performed a genetic screen for dominant mutations in the *PMS1* gene. We identified *pms1* null missense mutations that caused weakly dominant MMR defects when present in a wild-type *S. cerevisiae* strain on a single-copy plasmid. Interestingly, these mutations caused much stronger MMR defects when present on a single-copy plasmid in an *exo1Δ* mutant. Analysis of these mutations using the structure of the C-terminal domains of Mlh1-Pms1 [46] predicted that three amino acids altered by these mutations were metal ligands in the Mlh1-Pms1 nuclease active site and the fourth was a residue adjacent to the metal binding site. Biochemical analysis of mutant proteins confirmed that both *pms1* and *mlh1* mutations affecting the predicted active site eliminated or significantly reduced the RFC-PCNA dependent nuclease activity of Mlh1-Pms1 and *in vivo* imaging showed that the same mutations resulted in accumulation of Mlh1-Pms1-4GFP foci consistent with failure to execute a downstream step in MMR. These results both define the nuclease active site of the Mlh1-Pms1 endonuclease and thoroughly characterize the role of this endonuclease activity in a previously uncharacterized Exo1-independent MMR sub-pathway.

## Results

### Identification of dominant *pms1* mutations

To gain insight into the role of *PMS1* in mismatch repair, we sought to generate novel *pms1* mutations that cause a dominant MMR defect. First we mutagenized the *PMS1* gene by PCR amplification and gap-repaired the resulting DNA fragments into the low copy number *ARS CEN* pRS316 plasmid by co-transformation into a *S. cerevisiae* strain with a wild-type *PMS1* gene. A total of 38,000 transformants were screened for increased reversion of the *lys2-10A* frameshift mutation. This screen identified 211 transformants potentially containing dominant *pms1* mutations. Rescreening these 211 transformants for increased reversion of the *hom3-10* frameshift mutation identified 8 transformants with mutator phenotypes. The *pms1* mutation-bearing plasmids were isolated from these 8 transformants, and the *PMS1* gene from each plasmid was sequenced. Site directed mutagenesis and sub-cloning were used to construct *PMS1* plasmids containing single point mutations. These mutant plasmids were retested in the three mutator assays confirming four dominant *pms1* mutations resulting from amino acid substitutions in the C-terminal domain of Pms1: G683E, C817R, C848S, and H850R.

### Three dominant mutations eliminate metal binding ligands in the Pms1 endonuclease domain

**Figure 1:**
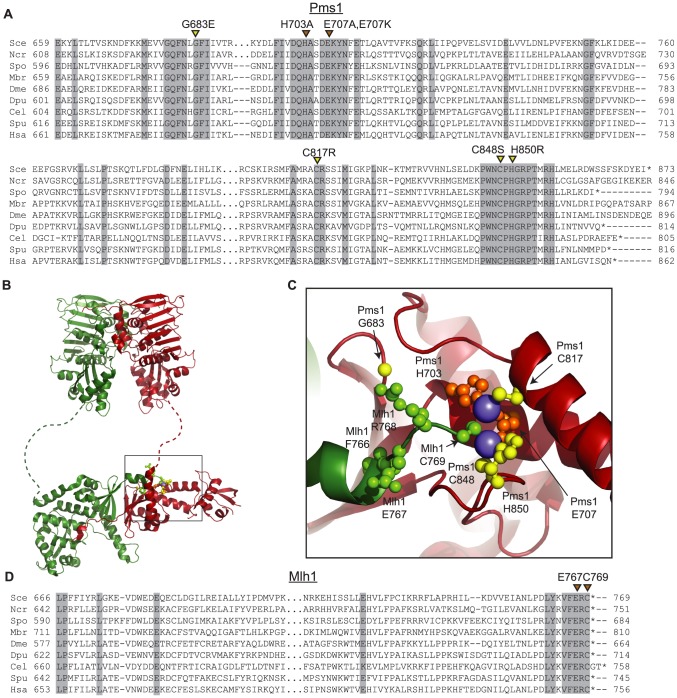
Conserved amino acid residues comprise the Mlh1-Pms1 endonuclease active site. **A.** Alignments of fungal Pms1 and animal Pms2 sequences from Sce (*Saccharomyces cerevisiae* NP_0141317.2), Ncr (*Neurospora crassa* XP_962690.2), Spo (*Schizosaccharomyces pombe* NP_594417.1), Mbr (Monosigna brevicollis XP_001747286.1), Dme (*Drosophila melanogaster* NP_477023.1), Dpu (*Daphnia pulex* EFX87911.1), Cel (*Caenorhabitis elegans* NP_505933.1), Spu (*Strongylocentrotus purpuratus* XP_786592.2), Hsa (*Homo sapiens* NP_000526.1) using Clustal Omega [72]. Asterisks indicate stop codons. Highlighted columns indicate perfectly conserved residues in the nine aligned species. Yellow triangles indicate the positions of mutations isolated in the screen. Orange triangles indicate the positions of engineered mutations generated in this study. **B.** Homology model of *S. cerevisiae* Mlh1-Pms1 with Mlh1 in green and Pms1 in red. The unstructured linker between the N- and C-terminal domains depicted as a dashed line. The position of the C-terminal active site is boxed. **C.** Modeled Pms1 endonuclease active site is displayed with the positions of amino acid substitutions isolated in the genetic screen shown with yellow ball-and-stick side chains and the position of the other metal ligands in orange. Position of the Mlh1 C-terminal FERC motif displayed as green ball-and-stick side chains. **D.** Alignments of fungal and animal Mlh1 sequences as in panel A from Sce (*Saccharomyces cerevisiae* NP_013890.1), Ncr (*Neurospora crassa* XP_962522.1), Spo (*Schizosaccharomyces pombe* NP_596199.1), Mbr (*Monosigna brevicollis*, reannotated starting from XP_001745742.1), Dme (*Drosophila melanogaster*, NP_477022.1), Dpu (*Daphnia pulex*, EFX86130.1), Cel (*Caenorhabitis elegans* NP_499796.2), Spu (*Strongylocentrotus purpuratus* XP_793318.2) and Hsa (*Homo sapiens* NP_000240.1).

The four amino acid substitutions resulting from the dominant mutations altered highly conserved amino acids (Figure 1A). Initial analysis of these amino acid substitutions using a homology model based on the C-terminal domains of endonuclease-proficient and zinc-binding *Neisseria gonorrhoeae* and *Bacillus subtilis* MutL homologs [47], [48] indicated that they affect the active site of the Pms1 endonuclease. Mapping of the amino acid substitutions onto the newly available structure of the C-terminal domains of Mlh1-Pms1 (Figure 1B,C) confirmed that the C817R, C848S, and H850R amino acid substitutions each eliminated one of the 5 metal ligands in the Pms1 endonuclease active site; all 5 ligands are conserved in eukaryotic ScPms1/HsPMS2 proteins and in the *N. gonorrhoeae* and *B. subtilis* MutL homologs [47], [48]. The fourth amino acid substitution, G683E, mapped to a conserved position adjacent to sites of metal coordination and could sterically disrupt the site or locally perturb the structure. We also constructed mutations resulting in the amino acid substitutions H703A and E707A to eliminate the remaining two predicted metal ligands not identified in our screen.

Fluctuation analysis was performed to evaluate the mutator effects of the dominant *pms1* mutants. Mutation rates were measured using the Can^R^ forward mutation assay and the *hom3-10* and *lys2-10A* frameshift reversion assays [21], [27] when the *pms1* dominant mutations were present on low copy plasmids in a strain with a wild-type *PMS1* gene (Table 1). None of the mutant plasmids caused more than a 2-fold increased mutation rate in the Can^R^ assay, which has a relatively high background mutation rate in wild-type cells and a low sensitivity for detecting MMR defects. In contrast, the mutations on the low copy plasmid caused between a 2- and 102-fold increase in mutation rate in the *hom3-10* and *lys2-10A* assays compared to introduction of a wild-type copy of *PMS1* on a low copy plasmid. Introduction of these mutations onto a high copy 2-micron plasmid resulted in much higher rates (Table S1), but still did not cause mutation rates that were as high as caused by deletion of *PMS1* (Compare Table S1 to Table S2). The *pms1* mutant plasmids were also unable complement a *pms1*Δ strain above that seen for the vector control (Table S2). Taken together, the fluctuation analysis indicated that the dominant *pms1* mutations were null *PMS1* alleles that caused a copy-number dependent dominant mutator phenotype. This dominant mutator phenotype was stronger for mutations causing amino acid substitutions of metal ligands as compared to the *pms1-G683E* mutation.

**Table 1:** Mutation rates of *pms1* metal coordination mutants on a low-copy plasmid in wild-type and *exo1Δ* strains.

		Mutation rate [95%CI] (fold increase relative to PMS1)*		
Plasmid Genotype	Yeast Genotype	Thr^+^	Lys^+^	Can^R^
*PMS1*	wild-type	7.31 [3.10–9.86]×10^−9^ (1)	2.12 [1.08–2.89]×10^−8^ (1)	1.58 [1.25–3.13]×10^−7^ (1)
EV	wild-type	7.58 [3.22–9.14]×10^−9^ (1)	2.12 [1.33–3.47]×10^−8^ (1)	2.34 [1.29–2.89]×10^−7^ (1.5)
*pms1H703A*	wild-type	6.59 [3.86–13.2]×10^−8^ (9)	7.02 [4.12–9.85]×10^−7^ (33)	2.66 [1.77–3.81]×10^−7^ (2)
*pms1E707A*	wild-type	3.52 [2.59–6.75]×10^−8^ (5)	4.18 [2.23–7.30]×10^−7^ (20)	2.13 [1.40–4.55]×10^−7^ (1)
*pms1G683E*	wild-type	1.64 [0.82–7.14]×10^−8^ (2)	3.74 [2.47–4.64]×10^−8^ (2)	1.33 [0.55–1.49]×10^−7^ (1)
*pms1C817R*	wild-type	4.67 [3.58–11.4]×10^−8^ (6)	1.51 [0.84–5.28]×10^−7^ (7)	3.50 [1.92–4.14]×10^−7^ (2)
*pms1C848S*	wild-type	3.24 [1.86–5.98]×10^−7^ (44)	2.17 [0.64–4.52]×10^−6^ (102)	4.52 [2.36–10.2]×10^−7^ (3)
*pms1H850R*	wild-type	3.13 [1.93–5.28]×10^−8^ (4)	1.99 [0.65–4.29]×10^−7^ (9)	2.48 [1.30–3.25]×10^−7^ (2)
*PMS1*	*exo1Δ*	2.13 [1.65–2.95]×10^−8^ (1)	7.62 [4.72–10.1]×10^−8^ (1)	9.19 [6.36–11.0]×10^−7^ (1)
EV	*exo1Δ*	2.45 [1.02–4.83]×10^−8^ (1)	1.62 [1.0–2.44]×10^−7^ (2)	2.78 [1.12–4.64]×10^−6^ (3)
*pms1H703A*	*exo1Δ*	1.47 [0.70–3.83]×10^−6^ (69)	1.40 [0.69–2.03]×10^−5^ (184)	1.79 [1.38–4.05]×10^−6^ (2)
*pms1E707A*	*exo1Δ*	1.22 [0.71–2.02]×10^−6^ (57)	9.39 [6.82–15.5]×10^−6^ (123)	1.74 [1.27–2.32]×10^−6^ (2)
*pms1G683E*	*exo1Δ*	3.36 [1.45–8.58]×10^−7^ (16)	2.75 [1.85–5.96]×10^−6^ (36)	1.66 [1.08–2.04]×10^−6^ (2)
*pms1C817R*	*exo1Δ*	2.47 [1.03–3.01]×10^−6^ (116)	7.31 [5.06–11.2]×10^−6^ (96)	2.46 [1.67–3.17]×10^−6^ (3)
*pms1C848S*	*exo1Δ*	3.57 [0.63–10.8]×10^−6^ (168)	1.60 [0.74–6.05]×10^−5^ (210)	2.83 [2.15–5.18]×10^−6^ (3)
*pms1H850R*	*exo1Δ*	1.94 [1.06–3.21]×10^−6^ (91)	7.09 [3.35–13.6]×10^−6^ (93)	1.68 [1.27–2.86]×10^−6^ (2)

* Median rates of hom3-10 (Thr^+^) and lys2-10A (Lys^+^) reversion and inactivation of CAN1 (Can^R^) with 95% confidence interval (CI) in square brackets and fold increase relative to complementation with pRS316-PMS1 in parentheses. For comparison, mutation rates for the *pms1Δ* strain complemented with empty vector (EV) are [Thr^+^ = 4.51×10^−5^, Lys^+^ = 2.77×10^−4^, Can^r^ = 9.61×10^−6^].

### Disruption of metal coordination inhibits endonuclease function

**Figure 2:**
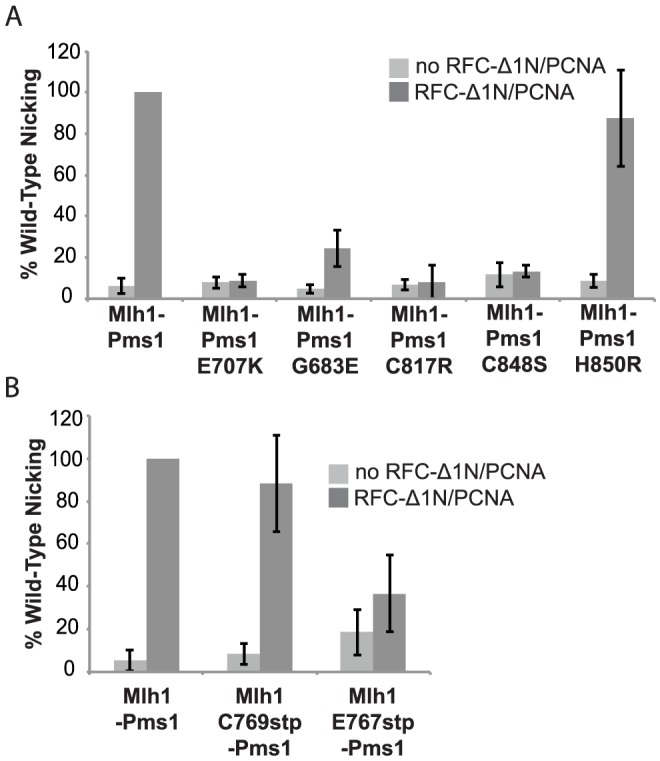
Metal coordination mutations eliminate the ability of Mlh1-Pms1 to nick closed circular DNA. Endonuclease reactions to nick closed circular DNA were performed with Mlh1-Pms1 alone or with Mlh1-Pms1, PCNA and RFC-Δ1N. **A.** Percentage of wild-type cleaved product formed by wild-type Mlh1-Pms1 and mutant Mlh1-Pms1 complexes containing the indicated Pms1 amino acid changes. **B.** Percentage of wild-type cleaved product formed by wild-type Mlh1-Pms1 and mutant Mlh1-Pms1 complexes containing the indicated Mlh1 amino acid changes. 100% cleavage of the 100 ng of pRS425 in the assay is 2.2 fmoles of cleavage events.

Previous studies have shown that *S. cerevisiae* Mlh1-Pms1 has a metal-dependent endonuclease activity that can be stimulated by RFC and PCNA [26]. To determine if the dominant *pms1* mutations affecting metal ligating amino acids disrupt the endonuclease function of Mlh1-Pms1, we expressed and purified the *S. cerevisiae* wild-type Mlh1-Pms1 complex and the mutant Mlh1-Pms1-G683E, Mlh1-Pms1-C817R, Mlh1-Pms1-C848S, and Mlh1-Pms1-H850R complexes and assayed the ability of these complexes to nick supercoiled pRS425 plasmid DNA with or without accessory factors RFC-Δ1N and PCNA (Figure 2A). Wild-type Mlh1-Pms1 alone showed little endonuclease activity. However, addition of PCNA and RFC-Δ1N to reactions containing wild-type Mlh1-Pms1 resulted in a 20-fold increase in endonuclease activity resulting in cleavage of nearly half of the original substrate DNA. The newly identified Mlh1-Pms1 mutant proteins and the previously studied Mlh1-Pms1-E707K mutant protein did not exhibit any PCNA and RFC-Δ1N stimulated endonuclease activity, with the exception of the Mlh1-Pms1-H850R mutant protein (Figure 2A). An explanation of the ability of the Mlh1-Pms1-H850R mutant protein to nick supercoiled DNA is provided in the “Discussion”. These results support the idea that loss of metal coordination by Pms1 inhibits the endonuclease activity of Mlh1-Pms1.

### *PMS1* metal coordination mutations cause a synthetic mutator phenotype when combined with an *exo1Δ* mutation

Exo1 is a 5′-3′ nuclease that functions in MMR *in vitro* by resecting DNA from a preexisting nick to a point past the mispair [28], [44], [49]. Loss of Exo1 function *in vivo* by deletion of *EXO1* or missense mutations inactivating the Exo1 active site only results in a weak mutator phenotype and hence only partial loss of MMR (Table 1) [27]–[29], [50]. To determine the consequences of eliminating the two known nucleases involved in *S. cerevisiae* MMR, we tested the effect of introducing plasmids containing the dominant *pms1* mutations into an *exo1Δ* mutant strain containing a wild-type *PMS1* gene. Relative to the effects in a wild-type strain, introduction of the dominant *pms1* mutations on a low-copy plasmid into the *exo1Δ* mutant strain increased the mutation rate to a much greater extent (Table 1). The *pms1-G683E* mutation again caused the weakest mutator phenotype, whereas the other five *pms1* mutations that affect the metal ligands caused relatively high mutation rates ranging from a 57- to a 210-fold increased mutation rate depending on the mutation and the assay. These results show that the dominant *pms1* mutations cause a greater MMR defect in an *exo1Δ* mutant strain than in a wild-type strain, suggesting that Exo1 and the Mlh1-Pms1 nuclease have a redundant function in MMR.

### Role of the conserved Mlh1 C-terminus in endonuclease activity and MMR

The four C-terminal residues of Mlh1 are almost completely conserved as the amino acids FERC in fungal and animal species (Figure 1D). Furthermore, Mlh1 has no C-terminal extension beyond the FERC residues in almost all sequenced eukaryotic organisms except nematodes (FERCG[T/S]) whereas the ScPms1/HsPMS2 family of proteins have variable length C-terminal extensions (Figure 1D). Consistent with a special role for the C-terminus of Mlh1, C-terminal fusions to the *S. cerevisiae* Mlh1 are non-functional for MMR *in vivo*, whereas C-terminal fusions to *S. cerevisiae* Pms1 do not affect function [38]. A structure of this highly conserved Mlh1 C-terminus (Figure 1B,C) revealed that this region might be appropriately positioned to play a role at the endonuclease active site of Pms1, with the C-terminal Mlh1 cysteine potentially acting as a metal ligand. We therefore generated the *mlh1-E767stp, mlh1-C769stp, mlh1-C769A* and *mlh1-C769S* mutations to probe the role of these highly conserved residues in endonuclease activity *in vitro* and MMR *in vivo*.

Unlike the *pms1* mutations affecting metal ligands, none of the mutations affecting the C of the conserved FERC motif of Mlh1 that is predicted to be a metal ligand including the *mlh1-C769A*, *mlh1-C769S*, and *mlh1-C769stp* mutations caused a dominant mutator phenotype when present on an *ARS CEN* plasmid in a wild-type strain or an *exo1Δ* strain (Table 2). The *mlh1-C769A*, *mlh1-C769S*, and *mlh1-C769stp* mutants fully complimented the MMR defect of an *mlh1Δ* strain (Table S2) consistent with previously published results for the *mlh1-C769A* mutation but not the *mlh1-C769S* mutation [51] or the *mlh1-C769stp* mutation [46]. In this regard, it should be noted that our studies used a broader series of mutator assays, including more sensitive assays, than previous studies of mutations affecting Mlh1-C767. In contrast, the *mlh1-E767stp* mutant plasmid failed to complement the MMR defect of an *mlh1Δ* strain and resulted in a null phenotype (Table S2). Furthermore, the *mlh1-E767stp* mutation on an *ARS CEN* plasmid caused a weak dominant mutator phenotype when present in a wild-type strain and a stronger dominant mutator phenotype when present in an *exo1Δ* strain (Table 2), although not to the extent as that caused by the *PMS1* metal ligand mutations.

**Table 2:** Mutations rates of MLH1-FERC motif mutations on a low-copy plasmid in wild-type and *exo1Δ* strains.

		Mutation rate [95%CI] (fold increase relative to MLH1)*		
Plasmid Genotype	Yeast Genotype	Thr^+^	Lys^+^	Can^R^
*MLH1*	wild-type	<1.35 [0.93–2.97]×10^−8^ (1.0)	3.47 [3.06–7.32]×10^−8^ (1.0)	2.36 [1.59–3.53]×10^−7^ (1.0)
EV	wild-type	7.58 [3.22–9.14]×10^−9^ (0.6)	2.12 [1.33–3.47]×10^−8^ (0.6)	2.34 [1.29–2.89]×10^−7^ (1.0)
*mlh1C769A*	wild-type	7.58 [5.53–14.2]×10^−9^ (0.6)	3.46 [2.14–5.21]×10^−8^ (1.0)	2.41 [1.51–2.91]×10^−7^ (1.0)
*mlh1C769S*	wild-type	9.43 [5.88–16.5]×10^−9^ (0.7)	2.64 [1.53–3.71]×10^−8^ (0.8)	2.98 [1.68–4.21]×10^−7^ (1.3)
*mlh1C769stp*	wild-type	1.17 [0.42–1.93]×10^−8^ (0.9)	2.20 [1.50–4.49]×10^−8^ (0.6)	2.53 [1.43–3.81]×10^−7^ (1.1)
*mlh1E767stp*	wild-type	1.32 [0.88–2.05]×10^−8^ (1.0)	1.38 [0.63–1.83]×10^−7^ (4.0)	2.59 [1.50–3.65]×10^−7^ (1.1)
*MLH1*	*exo1Δ*	3.13 [1.68–4.96]×10^−8^ (1.0)	2.45 [1.59–3.63]×10^−7^ (1.0)	1.20 [0.90–1.78]×10^−6^ (1.0)
EV	*exo1Δ*	2.45 [1.02–4.83]×10^−8^ (0.8)	1.62 [1.0–2.44]×10^−7^ (1.5)	2.78 [1.12–4.64]×10^−6^ (2.3)
*mlh1C769A*	*exo1Δ*	2.75 [1.47–7.58]×10^−8^ (0.9)	1.83 [1.40–2.65]×10^−7^ (0.7)	1.28 [0.96–1.56]×10^−6^ (1.2)
*mlh1C769S*	*exo1Δ*	3.53 [0.90–7.58]×10^−8^ (1.1)	1.28 [0.84–2.69]×10^−7^ (0.5)	1.03 [0.82–1.53]×10^−6^ (0.9)
*mlh1C769stp*	*exo1Δ*	4.24 [2.48–7.44]×10^−8^ (1.4)	1.32 [0.95–1.91]×10^−7^ (0.5)	1.08 [0.88–1.20]×10^−6^ (0.9)
*mlh1E767stp*	*exo1Δ*	1.75 [0.81–5.26]×10^−7^ (5.6)	1.66 [1.01–2.30]×10^−6^ (6.8)	1.55 [0.93–2.23]×10^−6^ (1.3)

* Median rates of hom3-10 (Thr^+^) and lys2-10A (Lys^+^) reversion and inactivation of CAN1 (Can^R^) with 95% confidence interval (CI) in square brackets and fold increase relative complementation with pRS316-MLH1 in parentheses. For comparison, mutation rates for the *mlh1Δ* strain complemented with empty vector (EV) are [Thr^+^ = 8.88×10^−5^, Lys^+^ = 2.23×10^−4^, Can^r^ = 9.85×10^−6^].

We also tested the effect of the *mlh1-C769stp* and *mlh1-E767stp* mutations in the endonuclease assay (Figure 2B). The mutant Mlh1-Pms1 protein lacking only the Mlh1 C-terminal cysteine that did not cause an MMR defect *in vivo* nicked supercoiled DNA to the same extent as the wild-type Mlh1-Pms1 protein. In contrast, the mutant Mlh1-Pms1 protein resulting from the *mlh1-E767stp* mutation was significantly defective for nicking supercoiled plasmid DNA, which parallels the effect of this mutation on MMR *in vivo*. These results support the idea that the C-terminus of Mlh1 functions in the endonuclease active site although if the terminal cysteine coordinates bound metal then this role is not required for MMR or endonuclease activity (Figure 1C).

### Mlh1-Pms1 metal coordination mutants do not preferentially affect MMR on the leading or lagging DNA strands

Mutations affecting the active sites of leading and lagging strand DNA polymerases Pol ε, *pol2-M644G*, and Pol δ, *pol3-L612M*, have been identified that preferentially introduce misincorporation errors in their respective strand during DNA replication [52], [53]. In a wild-type background, these lesions are then efficiently corrected by MMR. This strand-biased misincorporation can be used to determine strand preferences for MMR [52], [54], [55]. Here we probed mutants containing these polymerase active site mutations with *ARS CEN* plasmids encoding endonuclease defective *pms1* and *mlh1* mutations to investigate whether the Mlh1-Pms1 endonuclease preferentially functions in leading or lagging strand MMR. We found that *ARS CEN PMS1* plasmids containing the *pms1-C817R, pms1-C848S* or *pms1-H850R* mutations caused a statistically similar synergistic increase in mutation rate when present in strains containing mutations affecting either DNA polymerase (Table 3). Of nine pairwise comparisons between *pol2-M644G* and *pol3-L612M* mutants containing the same *pms1* mutation on a low copy plasmid using three different mutation rate assays, seven were not different (p-value>0.05, Mann-Whitney test). For the two comparisons that showed a difference, one showed a modestly higher rate in the *pol2-M644G* strain while the other showed a modestly higher rate in the *pol3-L612M* strain (p-value<0.05, Mann-Whitney test). This degree of similarity between the *pol2-M644G* and *pol3-L612M* mutants in these comparisons is in marked contrast to the effect of an *exo1Δ* mutation that caused a 9-fold higher increase in mutation rate in a *pol3-L612M* mutant compared to a *pol2-M644G* mutant [38]. Overall, these results suggest that Mlh1-Pms1 functions similarly on both the leading and lagging strands during MMR.

**Table 3:** Mutations rates of *pms1* metal coordination mutants on a low-copy plasmid in polymerase mutant strains *pol2M644G* and *pol3L612M*.

		Mutation rate [95%CI] (fold increase relative to PMS1)*		
Plasmid Genotype	Yeast Genotype	Thr^+^	Lys^+^	Can^R^
*PMS1*	*pol2M644G*	6.99 [3.43–10.8]×10^−8^ (1.0)	1.69 [0.73–2.25]×10^−7^ (1.0)	9.84 [4.99–32.4]×10^−6^ (1.0)
EV	*pol2M644G*	1.03 [0.52–5.62]×10^−7^ (1.4)	3.49 [2.15–5.21]×10^−7^ (2.1)	1.93 [0.52–6.15]×10^−5^ (2.0)
*pms1C817R*	*pol2M644G*	2.45 [0.73–11.9]×10^−7^ (3.5)†	1.44 [0.56–2.28]×10^−6^ (8.5)‡	4.23 [1.66–12.0]×10^−6^ (0.43)†
*pms1C848S*	*pol2M644G*	1.92 [0.79–9.54]×10^−6^ (27)†	1.70 [0.06–3.6]×10^−5^ (100)†	5.18 [1.62–96.6]×10^−6^ (0.53)†
*pms1H850R*	*pol2M644G*	1.08 [3.19–24.4]×10^−7^ (1.5)‡	2.19 [0.71–6.91]×10^−7^ (1.3)†	2.14 [1.04–6.77]×10^−6^ (0.22)†
*PMS1*	*pol3L612M*	5.79 [3.96–22.0]×10^−8^ (1.0)	1.10 [0.59–1.87]×10^−7^ (1.0)	3.78 [1.96–8.20]×10^−6^ (1.0)
EV	*pol3L612M*	7.42 [5.29–10.6]×10^−8^ (1.2)	1.04 [0.72–2.14]×10^−7^ (0.9)	3.66 [2.20–4.67]×10^−6^ (1.0)
*pms1C817R*	*pol3L612M*	3.38 [1.47–8.49]×10^−7^ (5.8)†	3.14 [2.15–6.62]×10^−7^ (2.9)‡	2.71 [1.22–13.6]×10^−6^ (0.7)†
*pms1C848S*	*pol3L612M*	1.43 [0.35–8.19]×10^−6^ (25)†	3.14 [0.11–10.7]×10^−6^ (29)†	9.09 [0.84–32.9]×10^−6^ (2.4)†
*pms1H850R*	*pol3L612M*	2.76 [1.06–7.63]×10^−7^ (4.8)‡	2.29 [1.39–6.09]×10^−7^ (2.1)†	1.96 [1.51–3.48]×10^−6^ (0.5)†

* Median rates of hom3-10 (Thr^+^) and lys2-10A (Lys^+^) reversion and inactivation of CAN1 (Can^R^) with 95% confidence interval (CI) in square brackets and fold increase relative to complementation with pRS316-PMS1 in parentheses.

† p-value>0.05 for a difference in mutation rate of the *pol2M644G* mutant compared to the *pol3L612M* mutant when both mutants contained the same indicated *pms1* mutant plasmid (Mann-Whitney test).

‡ p-value<0.05 for a difference in mutation rate of the *pol2M644G* mutant compared to the *pol3L612M* mutant when both mutants contained the same indicated *pms1* mutant plasmid (Mann-Whitney test).

### Mutations that affect Mlh1-Pms1 endonuclease function exhibit increased levels of Pms1 foci

**Figure 3:**
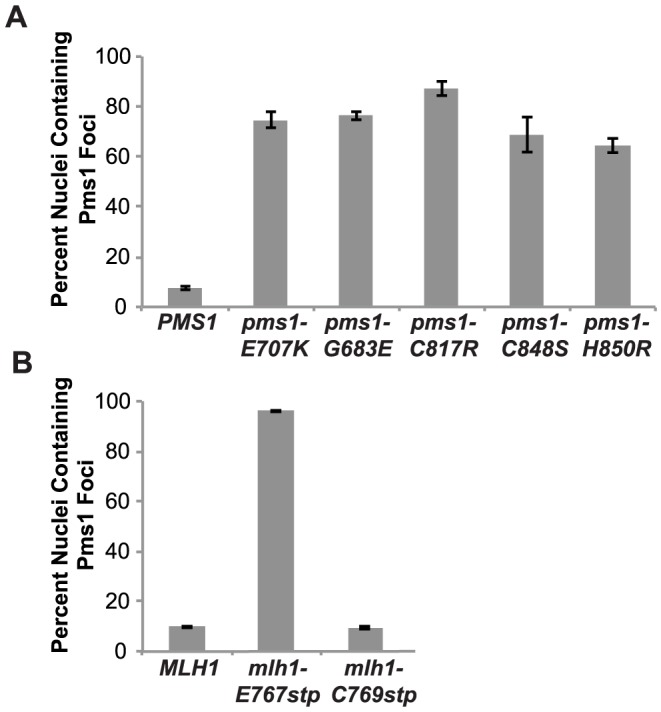
Zinc Metal coordination mutations cause increased levels of Mlh1-Pms1-4GFP foci. **A.** C-terminally tagged Pms1-GFP was imaged in logarithmically growing asynchronous cultures of the wild-type and the indicated *pms1* mutant strains. The quantitative data presented here, is the result of at least two independent experiments, each performed using two independent strain isolates. The total number of cells/nuclei (n) analyzed for each strain is indicated, and bars indicate the error of the mean (SEM). **B.** C-terminally tagged Pms1-GFP was imaged in logarithmically growing asynchronous cultures of wild-type and the indicated *mlh1-FERC* mutant strains as described under A.

We have previously demonstrated that Mlh1-Pms1 foci are an intermediate in MMR and that blocking MMR downstream of Mlh1-Pms1 recruitment results in increased levels of Mlh1-Pms1 foci [38]. To test the effect of the Mlh1-Pms1 active site mutations on the levels of Pms1 foci, different *mlh1* and *pms1* mutations were introduced into the relevant endogenous locus in a strain in which the single wild-type copy of *PMS1* was functionally tagged with four tandem copies of GFP. Normally, Mlh1-Pms1-4GFP foci are present in approximately 10% of logarithmically growing wild-type cells whereas all of the mutations that affected endonuclease function *in vitro* that were tested caused increased Mlh1-Pms1-4GFP foci formation (Figure 3). These included the metal coordination *pms1* mutations *pms1-E707K*, which was previously tested [38], as well as *pms1-C817R*, *pms1-C848S* and *pms1-H850R*, the *pms1-G683E* mutation and the *mlh1-E767stp* endonuclease active site mutation, which increased the proportion of cells containing Mlh1-Pms1-4GFP foci to between 64 and 96%. In contrast, the endonuclease and MMR proficient *mlh1-C769stp* mutation did not alter the levels of Mlh1-Pms1-4GFP foci compared to wild-type strain. These results are consistent with the idea that mispairs are recognized normally in these Mlh1-Pms1 endonuclease active site mutants and that there is proper loading of the mutant Mlh1-Pms1 on DNA but instead there is a mispair processing defect resulting in decreased turnover of the mutant Mlh1-Pms1 from the DNA [38].

## Discussion

In this study, we used the highly sensitive *lys2-10A* frameshift mutation reversion assay to screen mutagenized low copy *PMS1* plasmids for *pms1* mutations that caused a dominant mutator phenotype in the presence of a single-copy of the wild-type *PMS1* gene. We identified four null mutations that caused a weak dominant phenotype under these conditions. These mutations all caused amino acid substitutions in or near the region predicted to contain the Pms1 endonuclease active site and three of the amino acid substitutions including Pms1-C817R, Pms1-C848S, and Pms1-H850R affected predicted metal binding motifs while Pms1-G683 was in close proximity to the metal coordination site. The weak effect of these mutations explains why a prior study of a predicted Pms1 active site mutation did not observe a dominant effect [56]. A model of the endonuclease active site predicted Pms1 amino acids H703 and E707 as well as Mlh1-C769 to also be a part of the Mlh1-Pms1 active site [46]. Consistent with this model, the *pms1-H703A* and *pms1-H707A* mutations were found to cause the same phenotypes as the other *PMS1* metal ligand mutations. In contrast, no mutation affecting Mlh1-C769 caused either a dominant mutator phenotype or affected Mlh1 function whereas the *mlh1-E767stp* mutation caused phenotypes that were similar to those caused by the *PMS1* metal ligand mutations. Remarkably, all of the *pms1* mutations caused a stronger dominant mutator phenotype when present in an *exo1Δ* strain on a low copy plasmid. The *mlh1-E767stp* also caused an increased dominant mutator affect in the *exo1Δ* strain, although not to the extent seen with the *pms1* mutations. The phenotype of these mutants is similar to previously described separation-of-function mutations in *MSH2*, *MSH3*, *MSH6*, *MLH1*, *PMS1*, *POL30* and *POL32* that cause strong defects in Exo1-independent MMR but little if any defect in MMR when Exo1 is functional [27].

The asymmetry of the endonuclease active site is consistent with proposed roles of Mlh1-Pms1 in nicking double-stranded DNA during MMR; however, this asymmetry is not present in homodimeric bacterial MutL homologs with endonuclease function [47], [48]. The similarity of the eukaryotic Mlh1-Pms1 and bacterial MutL homologs suggests that MutL-DNA complexes may be functionally asymmetric so that only one active site is positioned to cleave DNA, analogous to the functional asymmetry during mispair recognition by bacterial MutS homodimers [15], [16]. The asymmetry of the eukaryotic MutL complexes, however, allows specialization of each subunit. The highly conserved C-terminus of Mlh1 is positioned in the Mlh1-Pms1 structure in a way that suggests that the Mlh1-ScPms1/HsPMS2 and Mlh1-Mlh3 complexes have a composite endonuclease active site. Potential roles for residues in the Mlh1 C-terminus include coordinating DNA phosphates, promoting nucleophilic attack by a water molecule or stabilization of the Pms1 active site. Consistent with this, the *mlh1-E767stp* mutant plasmid did not complement the mutator phenotype caused by a deletion of *MLH1*, and the *mlh1-E767stp* mutation resulted in the accumulation of Mlh1-Pms1-4GFP foci and reduced endonuclease activity similar to mutations in *PMS1* affecting the endonuclease active site. It was surprising that mutation or deletion of the highly conserved C-terminal cysteine did not cause a MMR defect *in vivo* or reduce endonuclease activity *in vitro* given the possibility that this residue might coordinate with metals in the endonuclease active site. It is possible that conservation of the C-terminal cysteine may reflect other roles for Mlh1, potentially including crossover resolution during meiosis [57]–[59].

Analysis of the genetically identified and structure-based mutations in the *MLH1* and *PMS1* genes revealed that disruption of the metal binding sites leads to disruption of the Mlh1-Pms1 endonuclease activity and arrest of MMR repair at a step following recruitment of Mlh1-Pms1 into microscopically-observable foci. In a Mn^2+^-, RFC-, and PCNA-dependent endonuclease assay, amino acid substitutions affecting all of the metal ligands caused defects in the Mlh1-Pms1 endonuclease activity, which confirms and extends previous studies of the human Mlh1-Pms2 E705K amino acid substitution [25], [26]. The *pms1-H850R* and the *mlh1-E767stp* mutations resulted in proteins with partial defects in the *in vitro* endonuclease assay, but caused complete MMR defects *in vivo*. The partial endonuclease defect caused by these mutations may reflect the fact that the *in vivo* metal ion in eukaryotic and bacterial homologs is Zn^2+^, which has tetrahedral coordination geometry, whereas the metal that promotes the *in vitro* assay is Mn^2+^, which prefers an octahedral coordination geometry [25], [48], [60]–[63]. All of the *pms1* mutations that affect predicted metal binding ligands, as well as *mlh1-E767stp*, caused complete MMR defects *in vivo*, consistent with the observation that metal ligand defects in human Mlh1-Pms2 inactivate MMR *in vitro* [26], [63]. These mutations also caused an accumulation of Mlh1-Pms1-4GFP foci indicating the step at which these mutations disrupt MMR is after loading of Mlh1-Pms1 by Msh2-Msh6, suggesting that loss of endonuclease activity leads to a turnover defect of Mlh1-Pms1 during MMR.

**Figure 4:**
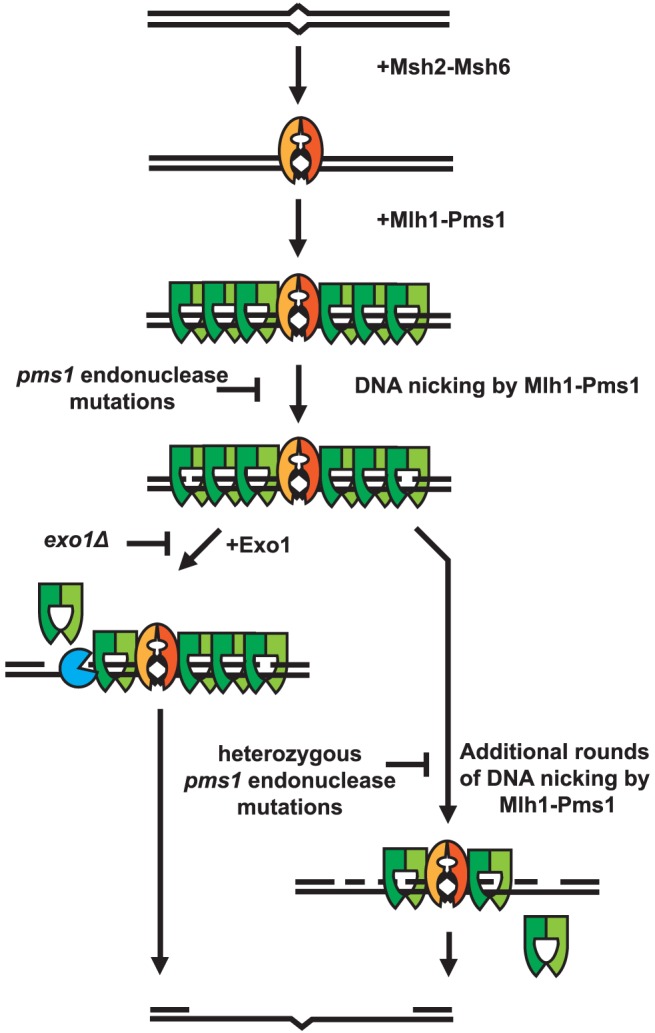
Model of Exo1-dependent and Exo1-independent resection of mispaired DNA. Mispaired DNA is recognized by the Msh2-Msh6 heterodimer and subsequently recruits super-stoichiometric amounts of Mlh1-Pms1. In the absence of endonuclease function, Mlh1-Pms1 has slow or no turnover and persistently remains on damaged DNA. When the Mlh1-Pms1 endonuclease is functional, it can introduce nicks into the DNA and either work in conjunction with Exo1 or function alone to resect past damaged DNA allowing for repair of DNA damage.

A striking property of the dominant *pms1* and *mlh1* mutations is that when present on a low copy plasmid they cause a much greater defect in Exo1-independent MMR compared to MMR when Exo1 is functional even though Mlh1-Pms1 appears to be absolutely required for all MMR. This phenotype is similar to the phenotype caused by the previously described separation-of-function mutations in genes like *MSH2*, *MSH3*, *MSH6*, *MLH1*, *PMS1*, *POL30* and *POL32* that result in strong defects in Exo1-independent MMR but little if any defect in MMR when Exo1 is functional [27]. A hypothesis that could explain these observations is that Mlh1-Pms1 has two roles in MMR, one involving activation of the Mlh1-Pms1 endonuclease and one where Mlh1-Pms1 plays a role in the recruitment of downstream MMR factors. It was previously shown that Exo1 interacts with Mlh1 and that this interaction is required for Exo1 to function in MMR [50]. This suggests the possibility that mispair recognition by Msh2-Msh6 or Msh2-Msh3 recruits Mlh1-Pms1 which then targets Exo1 to DNA where it could promote excision at pre-existing nicks in the DNA, consistent with the observation that lagging strand MMR is more Exo1 dependent than leading strand MMR [38], [42]. Such a reaction would be expected to be relatively insensitive to inhibition by competition with an endonuclease inactive but structurally normal form of Mlh1-Pms1 that would still bind Exo1 and target it to the site of MMR. In contrast, MMR in the absence of Exo1 might be completely dependent on the Mlh1-Pms1 endonuclease activity. This reaction would be expected to be competed for and interfered with by the presence of endonuclease inactive but structurally normal form of Mlh1-Pms1. A model that summarizes these concepts is presented in Figure 4.

## Materials and Methods

### Strains and media

*S. cerevisiae* cells were grown in YEPD (1% yeast extract, 2% Bacto peptone and 2% dextrose with or without 2% Bacto agar) or SD (0.67% yeast nitrogen base and 2% dextrose with or without 2% Bacto agar) medium. SD medium was supplemented with the appropriate dropout mix of amino acids (USA Biological). The *S. cerevisiae* strains used in genetic experiments were derived from an S288c parental strain and the strains used for protein purification were derived from RDKY1293 or RDKY8053 (listed in Table S3). All strains were constructed using standard gene disruption and transformation procedures. *E. coli* strains were propagated in LB media (0.5% yeast extract, 1% tryptone, 0.5% NaCl, 50 µg/ml thymine with or without 2% Bacto agar) containing 100 µg/ml ampicillin as required.

### Plasmids and plasmid construction

All plasmids (listed in Table S4) were maintained in *E. coli* TOP 10F′. A pRS316 Amp^r^ *URA3 ARS-CEN PMS1* plasmid pRDK1667 was constructed by recombination *in vivo*. Briefly, *PMS1* was amplified from *S. cerevisiae* S288c chromosomal DNA using the primers 5′ACGACGGCCAGTGAATTGTAATACGACTCACTATAGGGCGAATTGGAGCTattgccaaacaggcaaagac that contains 50 bp of homology to pRS316 upstream of the multiple cloning site followed by 20 bp of homology to the *PMS1* genes 707 bp upstream of the promoter starting at chromosome XIV coordinate 472684 and 5′TTAACCCTCACTAAAGGGAACAAAAGCTGGGTACCGGGCCCCCCCTCGAGgcatacaagaaacaacgcga that contains 50 bp of homology to pRS316 encompassing the *Xho*I, *Dra*II, *Apa*I and *Kpn*I sites of the multiple cloning site followed by 20 bp at the 3′ end with homology to the *PMS1* genes 302 bp downstream of the stop codon from chromosome XIV coordinate 476314. The PCR product was mixed with an equimolar amount of pRS316 that had been linearized by digestion with *Sma*I and co-transformed into the wild-type *S. cerevisiae* strain RDKY3590. The transformants were selected on SC-uracil drop out plates, DNA was isolated from individual transformants, rescued by transformation into *E. coli* and sequenced. The plasmid selected for further use has a silent C3055T mutation. A pRS426 Amp^r^ *URA3* 2-micron *PMS1* plasmid, pRDK1689, was constructed by subcloning the *Xho*I to *Stu*I (*Stu*I cuts in the *URA3* gene) *PMS1* fragment from pRDK1667 into the *Xho*I to *Stu*I backbone of pRS426. The pRS316 Amp^r^ *URA3 ARS-CEN MLH1* plasmid pRDK1338 was from our laboratory collection and contains a *Sac*I to *Xho*I *MLH1* fragment inserted between the *Sac*I and *Xho*I sites of pRS316. The *MLH1* fragment starts at the native *Sac*I site 2281 bp upstream of the *MLH1* ATG and ends at an *Xho*I site inserted by PCR 121 bp downstream of the *MLH1* stop codon. The 2-micron Mlh1 and Pms1 over-expression plasmids pRDK573 *TRP1 GAL10-MLH1* and pRDK1099 *LEU2 GAL10-PMS1-FLAG* have been described previously [64]. Mutations were made in these plasmids using standard site-directed mutagenesis methods or by subcloning from a mutant plasmid and the resulting plasmids were verified by DNA sequencing. The *PMS1* mutant alleles *E707K*, *H850R*, *C848S*, *G683E* and *C817R* were introduced at the chromosomal locus using standard pop in/out techniques employing the integrative plasmids listed in Table S4. These integration plasmids were generated by subcloning the *Xho*I-*Stu*I fragment containing the *pms1* mutant sequence from their respective pRS316-*pms1* mutant series plasmids into the *Xho*I-*Stu*I sites of the pRS306 backbone and were linearized with *Blp*I prior to transformation for integration into the strains of interest. The *MLH1* mutant alleles *E767stp* and *C769stp* were introduced at the chromosomal locus using standard gene disruption employing an *HPH* disruption cassette generated by PCR such the upstream homology targeted the C-terminus of *MLH1* and contained the mutations needed to introduce the *E767stp* and *C769stp* alleles. All of the chromosomal *pms1* and *mlh1* mutations were verified by sequencing the entire *PMS1* or *MLH1* gene as relevant, which also ensured that no additional mutations were introduced during strain construction.

### Isolation of random mutations in the *PMS1* gene

Mutagenesis of the *PMS1* gene by PCR was performed essentially as previously described [65] with the following modifications. The primers used for PCR were those described above for amplification of *PMS1*. Ten PCR reactions were performed using Klentaq DNA polymerase and a *PMS1* gene containing plasmid pRDK433 from our laboratory collection as a template. The PCR reactions were pooled, aliquots of DNA were mixed with an equimolar amount of pRS316 that had been linearized by digestion with *Sma*I and co-transformed into the wild-type *S. cerevisiae* strain RDKY3590. The transformants were plated on SC-uracil drop out plates to select for transformants, which were then replica plated onto SC-uracil-lysine drop out plates to screen for colonies that had increased rates of reversion of the *lys2-A10* frameshift mutation. Candidate mutator mutants were retrieved from the uracil drop out plates, restreaked on SC-uracil drop out plates, patched in duplicate onto uracil drop out plates and replica plated onto threonine-uracil drop out plates to screen for patches that had increased rates of reversion of the *hom3-10* frameshift mutation. Plasmid DNA was isolated from each mutator mutant, transformed into *E. coli* TOP10F′ and sequenced. Individual mutations identified were then transferred to a new pRS316 *PMS1* plasmid pRDK1667 by either sub-cloning using appropriate restriction endonuclease cleavage sites or by site-directed mutagenesis and retested essentially as described for the initial screen above.

### Protein purification

*S. cerevisiae* Mlh1-Pms1 was purified from 2.2 L of culture of the overproduction strain RDKY7608 (RDKY1293 containing the 2-micron plasmids pRDK573 *TRP1 GAL10-MLH1* and pRDK1099 *LEU2 GAL10pr-PMS1-FLAG*) (Table S3) according to a previously published procedure [64], except with the following 6 modifications: (1) Cell growth and induction of Mlh1-Pms1 expression utilized a published lactate to galactose shift protocol according to previously published methods [66]; (2) 2 mM β-mercaptoethanol was substituted for the 1 mM DTT in the buffers used to run the Heparin and FLAG antibody columns whereas all other buffers contained 1 mM DTT; (3) After washing the Heparin column with Buffer A containing 200 mM NaCl, the proteins were eluted using a single step of 1 M NaCl in Buffer A; (4) The pooled Heparin column fractions were diluted with Buffer A to obtain a final NaCl concentration of 500 mM prior to being subjected to 3 cycles of binding and elution from the FLAG antibody column; (5) The SP Sepharose column fractions were diluted with Buffer A to a final NaCl concentration of 200 mM prior to being loaded onto a 1 ml HiTrap Q column (GE Healthcare) followed by elution with a 100 mM to 1 M linear NaCl gradient run in Buffer A; and (6) The HiTrap Q column fractions containing the Mlh1-Pms1 were concentrated and desalted using a Centraprep (Ultracel 30K) spin column. The resulting Mlh1-Pms1 was contained in 0.5 ml of Buffer A +100 mM NaCl, and was frozen in liquid nitrogen and stored at −80 C. The Mlh1-Pms1-E707K, Mlh1-Pms1-C817R, Mlh1-Pms1-C848S and Mlh1-Pms1-H850R proteins were purified using the overproduction strains RDKY7696, RDKY7756, RDKY7759 and RDKY7793 (Table S3). The Mlh1-C769stp-Pms1 and Mlh1-E767stp-Pms1 proteins were purified using the overproduction strains RDKY8055 and RDKY8057 for which the RDKY8053 host strain (Table S3) was a derivative of RDKY1293 containing a deletion of the *MLH1* gene. Because *mlh1-E767stp* allele does not compliment the *MLH1* deletion in the host, after the protein expression period, DNA was isolated from the culture, 20 independent *MLH1* and *PMS1* plasmids were rescued by transformation into *E. coli* and sequenced to ensure that no mutations had occurred in the expression plasmids. *S. cerevisiae* PCNA and RFC-Δ1N were purified exactly as described in published procedures [66]–[68]. All of the protein preparations used in these studies were greater than 98% pure as analyzed by SDS-PAGE.

### Mlh1-Pms1 endonuclease assay

Mismatch-independent endonuclease assays were performed as a modification of one used previously [26]. 40 µL reactions containing 1 mM MnSO_4_, 20 mM Tris pH 7.5, 0.5 mM ATP 0.2 mg/mL bovine serum albumin (BSA), 2 mM DTT and 100 ng pRS425 were incubated at 30°C for 30 minutes. Reactions were terminated by incubation at 55°C following introduction of SDS, EDTA, glycerol and proteinase K at concentrations of 0.1%, 14 mM, 8% and 0.5 ug/ml respectively. Mlh1-Pms1, PCNA, or RFC-Δ1N were diluted to the appropriate working concentrations with a buffer comprised of 10% glycerol, 200 mM NaCl, 2 mM DTT and 20 mM Tris pH 7.5. Following termination of the reaction the samples were electrophoresed on a 0.8% agarose gel, the gel was stained with ethidium bromide, extensively destained and then the bands were quantified using a BioRad ChemiDoc XP imaging system. Serial dilutions of *Xho*I linearized pRS425 ranging from 10–100 ng were used as a concentration standard for quantification.

### Determination of mutation rates

Mutation rates were determined by fluctuation analysis. A single colony was used to inoculate a culture that was then diluted and used for transformation with a selectable plasmid carrying the desired allele and transformed colonies were selected by growth for 3 days at 30°C on SC-uracil dropout plates. 7 independent colonies were used to inoculate individual overnight cultures containing 10 ml of SC-uracil dropout media. Following cell growth, appropriate dilutions of the cultures were plated onto SC –uracil, –uracil-lysine, -uracil-threonine, and -uracil-arginine+canavanine dropout plates. The resulting colonies counted after growth at 30°C for 3 days and the average mutation rate was calculated for each strain as described previously [21], [27]. Each experiment was performed independently up to 4 times.

### Molecular modeling

Site-directed mutagenesis was guided by a molecular model of the C-terminal domains of Mlh1-Pms1. The C-terminus of *S. cerevisiae* Mlh1 was modeled using Phyre, xfit, and CNS [69]–[71] starting from the crystal structure of the human Mlh1 C-terminal domain (PDB id 3rbn). The C-terminus of *S. cerevisiae* Pms1 was similarly modeled using the crystal structures of *N. gonorrhoeae* [PDB id 3ncv; [47]] and *B. subtilis* [PDB ids 3gab, 3kdg, 3kdk; [48]] MutL homologs. Subsequently, the amino acid substitutions studied were mapped onto the newly available structure of the C-terminal domains of *S. cerevisiae* Mlh1-Pms1 [PDB id 4e4w; [46]].

### Live-cell imaging and image analysis

For microscopy studies, the C-terminus of each PMS1 protein of interest was fluorescently tagged by targeting a 4GFP tag to the chromosomal locus so that the native promoter was intact and expression remained unaffected. Previous analysis of the tagged *PMS1* gene demonstrated that the 4GFP tag did not affect the biological activity of Pms1 [38]. Exponentially growing cultures were washed and resuspended in water, placed on minimal media agar pads, covered with a coverslip, and imaged on a Deltavision (Applied Precision) microscope with an Olympus 100× 1.35NA objective. Fourteen 0.5 µm z sections were acquired and deconvolved with softWoRx software. Further image processing, including maximum intensity projections and intensity measurements were performed using ImageJ.

## Supporting Information

Table S1 Mutation rates caused by *pms1* metal coordination mutations on a high-copy plasmid in wild-type and *exo1Δ* mutant *S. cerevisiae* strains.
(DOCX)

Table S2 Mutations rates caused by *pms1* and *mlh1* mutations in *pms1Δ* or *mlh1Δ* mutant *S. cerevisiae* strains.
(DOCX)

Table S3 *S. cerevisiae* strains used in the experiments presented.
(DOCX)

Table S4 Plasmids used in the genetics and protein purification experiments presented.
(DOCX)
