# Supplementary material for: Dominant Mutations in S. cerevisiae PMS1 Identify the Mlh1-Pms1 Endonuclease Active Site and an Exonuclease 1-Independent Mismatch Repair Pathway
Source: PLoS Genet. 2013 Oct 31;9(10):e1003869. doi: 10.1371/journal.pgen.1003869 (PMC3814310; doi:10.1371/journal.pgen.1003869)
Supplement: Table S1 — Mutation rates caused by pms1 metal coordination mutations on a high-copy plasmid in wild-type and exo1Δ mutant S. cerevisiae strains. (DOCX) [file pgen.1003869.s001.docx]

|  |  | **Mutation rate [95%CI] (fold increase relative to PMS1)*** | | |
| --- | --- | --- | --- | --- |
| **Plasmid Genotype** | **Yeast Genotype** | **Thr^+^** | **Lys^+^** | **Can^R^** |
| *PMS1* | wild-type | 1.13 [0.74-5.52]x10^-8^ (1) | 2.24 [1.52-3.05]x10^-7^ (1) | 2.09 [1.87-5.07]x10^-7^ (1) |
| EV | wild-type | 7.58 [3.22-9.14]x10^-9^ (0.7) | 2.12 [1.33-3.47]x10^-8^ (0.1) | 2.34 [1.29-2.89]x10^-7^ (1.1) |
| *pms1G683E* | wild-type | 4.37 [2.97-6.65]x10^-6^ (387) | 2.50 [0.45-5.29]x10^-5^ (112) | 2.07 [1.83-2.74]x10^-6^ (10) |
| *pms1C817R* | wild-type | 4.54 [3.63-9.51]x10^-6^ (402) | 2.07 [0.73-5.55]x10^-5^ (92) | 2.11 [1.29-3.69]x10^-6^ (10) |
| *pms1C848S* | wild-type | 8.27 [4.19-13.6]x10^-6^ (732) | 5.17 [3.63-7.57]x10^-5^ (231) | 3.25 [2.60-4.39]x10^-6^ (16) |
| *pms1H850R* | wild-type | 1.12 [0.63-89.1]x10^-5^(991) | 5.82 [4.94-10.8]x10^-5^ (259) | 4.21 [2.87-10.2]x10^-6^ (20) |
| *PMS1* | *exo1Δ* | 1.19 [0.54-1.67]x10^-7^ (1) | 8.34 [5.30-11.5]x10^-7^ (1) | 8.76 [7.41-13.9]x10^-7^ (1) |
| EV | *exo1Δ* | 2.45 [1.02-4.83]x10^-8^ (0.2) | 1.62 [1.0-2.44]x10^-7^ (0.2) | 2.78 [1.12-4.64]x10^-6^ (3) |
| *pms1G683E* | *exo1Δ* | 2.05 [0.96-5.56]x10^-5^ (172) | 4.33 [0.54-7.05]x10^-5^ (52) | 5.99 [3.24-7.44]x10^-6^ (7) |
| *pms1C817R* | *exo1Δ* | 1.94 [1.08-5.20]x10^-5^(163) | 4.77 [2.64-8.51]x10^-5^(57) | 7.00 [3.78-8.94]x10^-6^(8) |
| *pms1C848S* | *exo1Δ* | 1.29 [0.99-4.24]x10^-5^(108) | 2.07 [0.16-3.82]x10^-5^(25) | 6.45 [4.04-12.0]x10^-6^(7) |
| *pms1H850R* | *exo1Δ* | 1.64 [0.72-3.95]x10^-5^(138) | 3.60 [2.02-7.01]x10^-5^(43) | 6.83 [3.24-12.6]x10^-6^ (8) |

**Table S1:** Mutation rates caused by *pms1* metal coordination mutations on a high-copy plasmid in wild-type and *exo1Δ* strains

* Median rates of hom3-10 (Thr^+^) and lys2-10A (Lys^+^) reversion and inactivation of CAN1 (Can^R^) with 95% confidence interval (CI) in square brackets and fold increase relative to complementation with pRS426-PMS1 in parentheses.
