# Supplementary material for: Dominant Mutations in S. cerevisiae PMS1 Identify the Mlh1-Pms1 Endonuclease Active Site and an Exonuclease 1-Independent Mismatch Repair Pathway
Source: PLoS Genet. 2013 Oct 31;9(10):e1003869. doi: 10.1371/journal.pgen.1003869 (PMC3814310; doi:10.1371/journal.pgen.1003869)
Supplement: Table S2 — Mutations rates caused by pms1 and mlh1 mutations in pms1Δ or mlh1Δ mutant S. cerevisiae strains. (DOCX) [file pgen.1003869.s002.docx]

|  |  | **Mutation rate [95%CI] (fold increase relative to PMS1)*** | | |
| --- | --- | --- | --- | --- |
| **Plasmid Genotype** | **Yeast Genotype** | **Thr^+^** | **Lys^+^** | **Can^R^** |
| *PMS1* | *pms1Δ* | 1.02 [0.82-2.03]x10^-8^ (1) | 5.77 [2.58-8.32]x10^-8^ (1) | 3.88 [1.68-5.64]x10^-7^ (1) |
| EV | *pms1Δ* | 4.51 [2.17-10.6]x10^-5^ (4421) | 2.77 [2.27-3.58]x10^-4^ (4800) | 9.61 [6.52-11.8]x10^-6^ (25) |
| *pms1C817R* | *pms1Δ* | 5.42 [3.20-11.0]x10^-5^ (5314) | 3.70 [2.40-5.00]x10^-4^ (6412) | 1.62 [1.13-2.27]x10^-5^ (42) |
| *pms1C848S* | *pms1Δ* | 5.52 [2.85-8.37]x10^-5^ (5412) | 2.28 [1.72-3.45]x10^-4^ (3951) | 1.46 [0.94-2.29]x10^-5^ (38) |
| *pms1H850R* | *pms1Δ* | 4.21 [2.69-6.68]x10^-5^ (4127) | 2.77 [1.77-3.18]x10^-4^ (4801) | 1.38 [0.78-2.88]x10^-5^ (36) |
| *MLH1* | *mlh1Δ* | 2.05 [0.71-7.34]x10^-7^ (1.0) | 5.36 [4.02-8.83]x10^-6^ (1.0) | 2.13 [1.62-3.99]x10^-7^ (1.0) |
| EV | *mlh1Δ* | 8.88 [3.68-12.6]x10^-5^ (433) | 2.23 [1.33-3.03]x10^-4^ (42) | 9.85 [8.08-15.9]x10^-6^ (46) |
| *mlh1C769A* | *mlh1Δ* | 2.97 [1.28-8.33]x10^-7^ (1.4) | 1.99 [1.72-3.17]x10^-6^ (0.4) | 2.84 [1.98-3.79]x10^-7^ (1.3) |
| *mlh1C769S* | *mlh1Δ* | 1.07 [0.70-5.42]x10^-7^ (0.5) | 3.07 [1.66-4.50]x10^-6^ (0.6) | 2.88 [1.91-7.87]x10^-7^ (1.4) |
| *mlh1C769stp* | *mlh1Δ* | 5.40 [3.19-14.2]x10^-8^ (0.3) | 8.51 [3.80-18.2]x10^-7^ (0.2) | 1.98 [0.98-3.37]x10^-7^ (0.9) |
| *mlh1E767stp* | *mlh1Δ* | 3.80 [2.46-10.8]x10^-5^ (185) | 2.68 [1.71-3.78]x10^-4^ (50) | 1.27 [0.78-2.13]x10^-5^ (60) |

**Table S2:** Mutations rates caused by *pms1* and *mlh1* mutations in *pms1Δ* or *mlh1Δ* strains

* Median rates of hom3-10 (Thr^+^) and lys2-10A (Lys^+^) reversion and inactivation of CAN1 (Can^R^) with 95% confidence interval (CI) in square brackets and fold increase relative to complementation with either pRS316-PMS1 or pRS316-MLH1 in parentheses.
