# Supplementary material for: Dominant Mutations in S. cerevisiae PMS1 Identify the Mlh1-Pms1 Endonuclease Active Site and an Exonuclease 1-Independent Mismatch Repair Pathway
Source: PLoS Genet. 2013 Oct 31;9(10):e1003869. doi: 10.1371/journal.pgen.1003869 (PMC3814310; doi:10.1371/journal.pgen.1003869)
Supplement: Table S3 — S. cerevisiae strains used in the experiments presented. (DOCX) [file pgen.1003869.s003.docx]

| **Name** | **Relevant genotype** | **reference** |
| --- | --- | --- |
| RDKY5964 | *MATa ura3-52 leu2Δ1 trp1Δ63 his3Δ200 hom3-10 lys2::InsE-A10* | Hombauer et al. *Cell* 2011 |
| RDKY7699 | RDKY5964 *msh3::HIS3 Msh6-mCherry.hphMX4 PMS1-4GFP.KanMX6* | Hombauer et al. *Cell* 2011 |
| RDKY7559 | RDKY5964 *msh3::HIS3 Msh6-mCherry.hphMX4 pms1-E707K-4GFP.KanMX6* | Hombauer et al. *Cell* 2011 |
| RDKY7885 | RDKY5964 *msh3::HIS3 Msh6-mCherry.hphMX4 pms1-H850R-4GFP.KanMX6* | this study |
| RDKY7886 | RDKY5964 *msh3::HIS3 Msh6-mCherry.hphMX4 pms1-C848S-4GFP.KanMX6* | this study |
| RDKY7887 | RDKY5964 *msh3::HIS3 Msh6-mCherry.hphMX4 pms1-G683E-4GFP.KanMX6* | this study |
| RDKY7888 | RDKY5964 *msh3::HIS3 Msh6-mCherry.hphMX4 pms1-C817R-4GFP.KanMX6* | this study |
| RDKY7565 | RDKY5964 *msh3::HIS3 PMS1-4GFP.KanMX6* | Hombauer et al. *Cell* 2011 |
| RDKY7889 | RDKY5964 *msh3::HIS3 mlh1-E767stp.hphNT1 PMS1-4GFP.KanMX6* | this study |
| RDKY7890 | RDKY5964 *msh3::HIS3 mlh1-C769stp.hphNT1 PMS1-4GFP.KanMX6* | this study |
| RDKY3686 | *Matα ura3-52 leu2Δ1 trp1Δ63 his3Δ200 hom3-10 lys2::InsE-A10* | Amin et al. *MCB* 2001 |
| RDKY4236 | *Mat*α *ura3-52 leu2*Δ*1 trp1*Δ*63 his3*Δ*200 hom3-10 lys2::InsE-A10 mlh1::hisG* | laboratory collection |
| RDKY4238 | *Mat*α *ura3-52 leu2*Δ*1 trp1*Δ*63 his3*Δ*200 hom3-10 lys2::InsE-A10 pms1::hisG* | laboratory collection |
| RDKY6974 | *Mat*α *ura3-52 leu2*Δ*1 trp1*Δ*63 his3*Δ*200 hom3-10 lys2::InsE-A10 exo1::hph* | this study |
| RDKY7537 | *Mat*α *ura3-52 leu2*Δ*1 trp1*Δ*63 his3*Δ*200 hom3-10 lys2::InsE-A10 pol2-M644G* | Hombauer et al. *Cell* 2011 |
| RDKY7538 | *Mat*α *ura3-52 leu2*Δ*1 trp1*Δ*63 his3*Δ*200 hom3-10 lys2::InsE-A10 pol3-L612M* | Hombauer et al. *Cell* 2011 |
| RDKY1293 | *Matα ura3-52 leu2Δ1 trp1 his3Δ200 pep4::HIS3 prb1Δ1.6R can1 GAL* | laboratory collection |
| RDKY7608 | RDKY1293 pRDK573 (*MLH1*) pRDK1099 (*PMS1*-FLAG) | this study |
| RDKY7694 | RDKY1293 pRDK573 (*MLH1*) pRDK1682 (*pms1G683E*-FLAG) | this study |
| RDKY7696 | RDKY1293 pRDK573 (*MLH1*) pRDK1686 (*pms1E707K*-FLAG) | this study |
| RDKY7756 | RDKY1293 pRDK573 (*MLH1*) pRDK1683 (*pms1C817R*-FLAG) | this study |
| RDKY7759 | RDKY1293 pRDK573 (*MLH1*) pRDK1684 (*pms1C848S*-FLAG) | this study |
| RDKY7993 | RDKY1293 pRDK573 (*MLH1*) pRDK1685 (*pms1C850R*-FLAG) | this study |
| RDKY8053 | *Matα ura3-52 leu2Δ1 trp1 his3Δ200 pep4::HIS3 prb1Δ1.6R can1 GAL mlh1::hph* | this study |
| RDKY8055 | RDKY8053 pRDK1680 (*mlh1C769stp*) pRDK1099 (*PMS1*-FLAG) | this study |
| RDKY8057 | RDKY8053 pRDK1681 (*mlh1E767stp*) pRDK1099 (*PMS1*-FLAG) | this study |

**Table S3:** *S. cerevisiae* strains

The references cited are as follows. Amin NS, Nguyen MN, Oh S, Kolodner RD (2001) exo1-Dependent mutator mutations: model system for studying functional interactions in mismatch repair. Mol Cell Biol 21: 5142-5155 and Hombauer H, Campbell CS, Smith CE, Desai A, Kolodner RD (2011) Visualization of eukaryotic DNA mismatch repair reveals distinct recognition and repair intermediates. Cell 147: 1040-1053.
