# Supplementary material for: Dominant Mutations in S. cerevisiae PMS1 Identify the Mlh1-Pms1 Endonuclease Active Site and an Exonuclease 1-Independent Mismatch Repair Pathway
Source: PLoS Genet. 2013 Oct 31;9(10):e1003869. doi: 10.1371/journal.pgen.1003869 (PMC3814310; doi:10.1371/journal.pgen.1003869)
Supplement: Table S4 — Plasmids used in the genetics and protein purification experiments presented. (DOCX) [file pgen.1003869.s004.docx]

| **Name** | **Relevant genotype** | **reference** |
| --- | --- | --- |
| pRDK573 | *amp^R^ ori 2µ TRP1 GAL10 MLH1* | Hargreaves et al. *JBC* 2010 |
| pRDK1099 | *amp^R^ ori 2µ LEU2 GAL10 PMS1-FLAG* | Hargreaves et al. *JBC* 2010 |
| pRDK1583 | *amp^R^ ori URA3 pms1E707K* | Hombauer et al. *Cell* 2011 |
| pRDK1659 | *amp^R^ ori URA3 pms1G683E* | this study |
| pRDK1660 | *amp^R^ ori URA3 pms1H850R* | this study |
| pRDK1661 | *amp^R^ ori URA3 pms1C848S* | this study |
| pRDK1662 | *amp^R^ ori URA3 pms1C817R* | this study |
| pRDK1667 | *amp^R^ CEN6 ARSH4 URA3 PMS1* | this study |
| pRDK1668 | *amp^R^ CEN6 ARSH4 URA3 pms1G683E* | this study |
| pRDK1669 | *amp^R^ CEN6 ARSH4 URA3 pms1C817R* | this study |
| pRDK1670 | *amp^R^ CEN6 ARSH4 URA3 pms1C848S* | this study |
| pRDK1671 | *amp^R^ CEN6 ARSH4 URA3 pms1H850R* | this study |
| pRDK1688 | *amp^R^ CEN6 ARSH4 URA3 pms1E707A* | this study |
| pRDK1687 | *amp^R^ CEN6 ARSH4 URA3 pms1H707A* | this study |
| pRDK1338 | *amp^R^ CEN6 ARSH4 URA3 MLH1* | this study |
| pRDK1674 | *amp^R^ CEN6 ARSH4 URA3 mlh1C769A* | this study |
| pRDK1675 | *amp^R^ CEN6 ARSH4 URA3 mlh1C769S* | this study |
| pRDK1676 | *amp^R^ CEN6 ARSH4 URA3 mlh1C769stp* | this study |
| pRDK1677 | *amp^R^ CEN6 ARSH4 URA3 mlh1E767stp* | this study |
| pRDK1689 | *amp^R^ ori 2µ URA3 PMS1* | this study |
| pRDK1690 | *amp^R^ ori 2µ URA3 pms1G683E* | this study |
| pRDK1691 | *amp^R^ ori 2µ URA3 pms1C817R* | this study |
| pRDK1692 | *amp^R^ ori 2µ URA3 pms1C848S* | this study |
| pRDK1693 | *amp^R^ ori 2µ URA3 pms1H850R* | this study |
| pRDK1678 | *amp^R^ ori 2µ TRP1 GAL10 mlh1C769A* | this study |
| pRDK1679 | *amp^R^ ori 2µ TRP1 GAL10 mlh1C769S* | this study |
| pRDK1680 | *amp^R^ ori 2µ TRP1 GAL10 mlh1C769stp* | this study |
| pRDK1681 | *amp^R^ ori 2µ TRP1 GAL10 mlh1E767stp* | this study |
| pRDK1682 | *amp^R^ ori 2µ LEU2 GAL10 pms1G683E-FLAG* | this study |
| pRDK1683 | *amp^R^ ori 2µ LEU2 GAL10 pms1C817R-FLAG* | this study |
| pRDK1684 | *amp^R^ ori 2µ LEU2 GAL10 pms1C848S-FLAG* | this study |
| pRDK1685 | *amp^R^ ori 2µ LEU2 GAL10 pms1H850R-FLAG* | this study |
| pRDK1686 | *amp^R^ ori 2µ LEU2 GAL10 pms1E707K-FLAG* | this study |

**Table S4:** Plasmids

The references cited are as follows. Hargreaves VV, Shell SS, Mazur DJ, Hess MT, Kolodner RD (2010) Interaction between the Msh2 and Msh6 nucleotide-binding sites in the Saccharomyces cerevisiae Msh2-Msh6 complex. J Biol Chem 285: 9301-9310 and Hombauer H, Campbell CS, Smith CE, Desai A, Kolodner RD (2011) Visualization of eukaryotic DNA mismatch repair reveals distinct recognition and repair intermediates. Cell 147: 1040-1053.
